# A mild form of a Stevens-Johnson syndrome to ciprofloxacin

**DOI:** 10.1186/2045-7022-4-S3-P93

**Published:** 2014-07-18

**Authors:** Elena Madalan, Gabriela Florescu, Petre Calistru

**Affiliations:** 1 Center for Diagnostic and Treatment "Dr. Victor Babes", Allergology and Clinical Immunology, Romania; 2 Center for Diagnostic and Treatment "Dr. Victor Babes", Dermatology, Romania; 3 Center for Diagnostic and Treatment "Dr. Victor Babes", Infectious diseases, Romania

## 

Ciprofloxacin is a widely used antibiotic with large spectrum activity both on Gram negative and Gram positive bacterial pathogens, valued for its rare incidence of hypersensitivity reactions. Delayed-type reactions are reported more often then the immediate-type. Herein we present the case of a 53 years old female patient who developed a symmetric maculopapular exanthematous rash, mainly on the trunk, in association with fever, dysphagia and oral mucosal involvement on the tenth day of ciprofloxacin therapy for recurrent urinary tract infection. The allergic history of the patient revealed two contact dermatitis episodes due to exposure to propolis. The clinical presentation as an early phase of a Stevens-Johnson syndrome was confirmed by the histological findings. The evolution of the cutaneous lesions was rapidly improved by oral corticosteroids and antihistamines. Due to the frequent use of the ciprofloxacin, the potential life-threatening hypersensitivity reactions should be taken into consideration.

